# Effect of *Pinus koraiensis* leaf extract on fatigue reduction and exercise performance: study protocol for a randomized, double-blind, placebo-controlled clinical trial

**DOI:** 10.3389/fmed.2025.1653559

**Published:** 2025-09-05

**Authors:** Yujin Choi, Changsop Yang, Ji Soo Yoon, Sunhoo Kim, Sang-Yoon Kim, Mi Young Lee

**Affiliations:** ^1^KM Science Research Division, Korea Institute of Oriental Medicine, Daejeon, Republic of Korea; ^2^Huons Dongam R&D Center, Huons N, Gwacheon, Republic of Korea; ^3^KM Convergence Research Division, Korea Institute of Oriental Medicine, Daejeon, Republic of Korea

**Keywords:** *Pinus koraiensis*, pine leaf extract, fatigue, exercise performance, dietary supplements, functional food, clinical trial protocol

## Abstract

**Background:**

Previous research has shown that *Pinus koraiensis* leaf (PKL) extract exhibits promising anti-fatigue potential in mice, showing improved exercise endurance, decreased stress-related biochemical markers, and enhanced antioxidant activity. Based on this previous animal study, the present study aims to evaluate the efficacy of PKL extract on fatigue reduction and exercise performance in healthy adults with moderate to severe fatigue.

**Method and analysis:**

This randomized, double-blind, placebo-controlled clinical trial will enroll volunteers who meet the eligibility criteria, specifically those with a Fatigue Severity Scale (FSS) score of 27 or higher, indicating clinically significant fatigue. Eligible participants will be randomly assigned to either the PKL extract or placebo group. The intervention will consist of daily consumption of the assigned investigational product for 8 weeks, with evaluations conducted at 4-weeks intervals. Primary outcome measures include the Fatigue Severity Scale (FSS) and time to exhaustion during a treadmill exercise test. Secondary outcomes include blood fatigue markers, exercise performance parameters, rating of perceived exertion, visual analogue scale for fatigue, and grip strength.

**Discussion:**

This clinical trial aims to develop PKL extract as a functional health food ingredient for fatigue improvement. The findings will provide evidence regarding the efficacy of PKL extract for alleviating fatigue and enhancing exercise performance in humans.

## 1 Introduction

Fatigue is a common subjective symptom (1), defined in dictionaries as extreme tiredness resulting from mental or physical exertion, which distinguishes it from tiredness or sleepiness in that it occurs due to excessive effort (2). Most fatigue is alleviated by sufficient rest; however, when it persists for more than 6 months, it is referred to as chronic fatigue (3), and when accompanied by symptoms such as cognitive impairment, sore throat, tender lymph nodes, muscle pain, joint pain, headaches, non-restorative sleep, and post-exertional malaise, it is classified as chronic fatigue syndrome (4). According to a recent meta-analysis, the prevalence of general fatigue (persisting less than 6 months) in the adult population was 20.4%, while the prevalence of chronic fatigue persisting for more than 6 months was 10.1% (3). There is growing interest in effective, safe, and accessible interventions to improve these fatigue symptoms, with particular attention being given to the development of functional food from natural substances.

The physiological mechanism of fatigue is closely linked to two metabolic pathways that produce energy during exercise: anaerobic and aerobic metabolism (5). During high-intensity exercise, muscles primarily rely on anaerobic metabolism, which leads to lactate accumulation and increased lactate dehydrogenase (LDH) activity. Lactate accumulation reduces muscle pH, impairing muscle contractile function and causing fatigue (6). Additionally, creatine kinase (CK) serves as an indicator of muscle damage, increases during exercise, and is associated with fatigue (7). During prolonged exercise, aerobic metabolism predominantly contributes, with glycogen depletion, increased production of reactive oxygen species (ROS), and decreased activity of antioxidant enzymes (SOD, GSH-Px) being associated with fatigue (8). Furthermore, increased levels of the stress hormone corticosterone and decreased serotonin are related to mental fatigue. These indicators play an important role in assessing fatigue status and anti-fatigue effects.

Meanwhile, recent research has suggested the potential anti-fatigue effects of *Pinus koraiensis* leaf extract (PKL). According to a single animal study by Lee et al. (9), PKL extract improved exercise endurance in mice during weight-loaded forced swimming and rotarod tests. In terms of biochemical markers, PKL extract significantly reduced blood lactate and LDH levels related to anaerobic metabolism, as well as decreased CK levels, an indicator of muscle damage. Regarding aerobic metabolism, PKL extract significantly increased the levels of antioxidant enzymes SOD and GSH-Px, which typically decrease after exercise, demonstrating a protective effect against oxidative stress. Additionally, PKL extract improved energy metabolism by increasing glycogen levels in both liver and muscle. These results suggest that PKL extract may exert anti-fatigue effects through metabolic regulation, and antioxidant activity. Based on these promising animal findings, whether these effects also occur in humans has not yet been explored.

Therefore, this study was designed to evaluate whether the anti-fatigue effects of PKL extract observed in previous animal experiments also occur in humans. Specifically, we aim to assess the anti-fatigue and exercise performance-enhancing effects of 8-weeks PKL extract consumption compared to placebo intake in healthy adults with high fatigue levels. If the efficacy of PKL extract in humans is confirmed through this study, it could be developed as a new functional food ingredient for fatigue improvement. This clinical trial was designed as a randomized controlled trial comparing the superiority of the PKL extract group over the placebo group, with parallel comparison between the two groups, and an allocation ratio of 1:1.

## 2 Methods and analysis

### 2.1 Study setting

This clinical trial is being conducted in an academic hospital, located in Jeonju, Republic of Korea.

### 2.2 Eligibility criteria

#### 2.2.1 Inclusion criteria

This clinical trial will enroll adult men and women aged 19–60 years at the time of screening who have a Fatigue Severity Scale (FSS) score of 27 or higher. All participants must be fully informed about the clinical trial, understand its details, agree to exercise tolerance testing, and voluntarily decide to participate and comply with precautions as documented by written informed consent.

#### 2.2.2 Exclusion criteria

Participants will be excluded from the clinical trial if they have abnormal BMI (<18.5 or ≥30 kg/m^2^), clinically significant acute or chronic diseases (cardiovascular, endocrine, immune, hepatobiliary, renal, urinary, neuropsychiatric, inflammatory, hematologic-oncologic, gastrointestinal, or musculoskeletal disorders), uncontrolled hypertension or diabetes (HbA1c > 7%), tuberculosis, multiple sclerosis, hypothyroidism, uncontrolled asthma, psychiatric disorders (major depressive disorder, bipolar disorder, schizophrenia, dementia, delusional disorder), insomnia, history of renal failure, heart failure, myocardial infarction, stroke, relevant gastrointestinal diseases or surgeries, myasthenia gravis, any paralytic or atrophic myopathy, inability to perform exercise, hypersensitivity to pine nuts or pine needle components, recent use of fatigue-related products (within 1 month), exercise supplements (within 2 weeks), or antipsychotic medications (within 2 months), alcohol/drug abuse, participation in another clinical trial within 3 months, abnormal laboratory values (AST/ALT > 3 × upper limit, serum creatinine > 2.0 mg/dL), pregnancy, lactation, refusal to use adequate contraception (if applicable), or if deemed unsuitable by the principal investigator.

### 2.3 Interventions

#### 2.3.1 PKL extract and placebo capsule

Participants will receive either PKL extract or placebo capsule. The PKL is derived from the leaf of *Pinus koraiensis* Siebold & Zucc. [Pinaceae]. Each PKL capsule (680 mg) contains 480 mg of PKL extract powder. The extract has been standardized based on lambertianic acid (10, 11) as the marker compound, with a concentration of 13.53 ± 0.41 mg/g of extract powder. The placebo capsule contains no active ingredients. Both the PKL extract and placebo capsules are white opaque hard capsules containing dark greenish-brown powder. Participants will take one capsule orally once daily after dinner.

The daily dose of 480 mg of PKL extract was calculated based on preclinical studies conducted with 7-weeks old male C57BL/6 mice (unpublished data). Anti-fatigue effects were confirmed at oral doses of 50 mg/kg, 100 mg/kg, and 200 mg/kg of PKL extract. Using 100 mg/kg body weight/day as the reference animal dose, the human equivalent dose was calculated as: 100 mg/kg (animal dose) × 3 (animal Km) ÷ 37 (human Km) = 8.10 mg/kg. For a 60 kg adult, this equates to 8.10 mg/kg × 60 kg = 486 mg, which was rounded to 480 mg for the final dosage.

#### 2.3.2 Criteria for discontinuing allocated interventions

The completion status of all participants will be recorded, with documentation of reasons for discontinuation. Study participation may be terminated if: major inclusion/exclusion violations are discovered after enrollment; the participant becomes pregnant; a serious adverse event occurs or the participant requests withdrawal due to an adverse event; previously undetected systemic disease is discovered; consent is withdrawn; major protocol violations occur; the participant is lost to follow-up; difficulty with investigational product consumption arises; prohibited medications affecting study outcomes are taken; or investigators determine continued participation is inappropriate.

#### 2.3.3 Procedure for monitoring adherence

Adherence to the investigational product consumption will be monitored during the second and third visits. Participants will be instructed to bring any remaining investigational product capsules with them to these visits. The investigators will collect the remaining capsules and verify adherence by counting them.

#### 2.3.4 Permitted and prohibited concomitant interventions

Medications taken for at least 1 month prior to enrollment may be permitted if deemed not to interfere with study outcomes, and temporary medications for other conditions may be allowed; however, any medication or health functional food potentially affecting efficacy evaluation will result in participant withdrawal. During the trial period, participants are prohibited from using health functional foods related to fatigue improvement [such as plum extract (12), Rhodiola rosea extract (13), ginseng extracts (14)], exercise performance enhancers [including creatine (15), Cordyceps extract (16)], performance enhancing supplements (protein supplements, multivitamins), and medications affecting fatigue or muscle strength.

### 2.4 Outcomes

#### 2.4.1 Primary outcomes

The Fatigue Severity Scale (FSS) scores change from baseline to 8 weeks is designated as the first primary outcome (17, 18). The FSS is a self-administered questionnaire consisting of 9 items that evaluate the subjective level of fatigue-related disability on a scale of 1–7. Scores below 27 indicate “low fatigue,” scores between 27 and 44 indicate “medium fatigue,” and scores of 45 or above indicate “high fatigue.” FSS will be measured at baseline, 4 weeks, and 8 weeks.

The second primary outcome is the change in time and distance to exhaustion during a treadmill exercise test from baseline to 8 weeks. The exercise test utilizes the Modified Bruce protocol, which progressively increases both speed and incline to measure exercise capacity (19). The test begins at stage 1 (2.7 km/h, 0% grade, 3 min) and continues through predefined stages of increasing difficulty (stage 2: 2.7 km/h, 5% grade, 3 min; stage 3: 2.7 km/h, 10% grade, 3 min; stage 4: 4.0 km/h, 12% grade, 3 min; stage 5: 5.4 km/h, 14% grade, 3 min; stage 6: 6.7 km/h, 16% grade, 3 min; stage 7: 8.0 km/h, 18% grade, 3 min). The test is terminated when the participant voluntarily requests to stop. The distance to exhaustion is calculated using the total exercise duration and speed. Treadmill exercise test will be conducted at baseline, and 8 weeks.

#### 2.4.2 Secondary outcomes

Secondary outcomes include blood fatigue markers (lactate, ammonia, inorganic phosphorus, LDH, CK, glucose, free fatty acid) (20, 21) measured at baseline and 8 weeks, with samples collected at rest, immediately after treadmill exercise test, and 30 min after recovery; exercise test parameters including anaerobic threshold (AT), maximal oxygen uptake (VO2max), maximal respiratory quotient (RQmax), and maximal heart rate (HRmax) during treadmill exercise test (19); rating of perceived exertion (RPE) assessed using Borg’s 15-point scale (6–20) (22) measured just before the end of each exercise test stage; visual analogue scale (VAS) for subjective fatigue evaluation on a 10 cm horizontal line (0–10); and hand grip strength of the dominant hand measured using a digital dynamometer in a seated position with the elbow flexed at 90 degrees, taking the highest value from three maximum grip efforts alternating between hands with 1-min intervals.

#### 2.4.3 Safety outcomes

Safety will be assessed through monitoring of subjective and objective adverse events, which will be collected through spontaneous reporting by participants, telephone follow-ups, and regular clinical evaluations during scheduled visits. Adverse event documentation will include onset and resolution dates, severity and outcome, actions taken regarding the investigational product, causality assessment, suspected concomitant medications, and any treatments administered. Laboratory tests will be conducted, including complete blood count (WBC, RBC, Hemoglobin, Hematocrit, Platelets), liver function tests (ALP, gamma-GT, AST, ALT, total bilirubin, total protein, albumin), kidney function tests (BUN, creatinine), lipid profile (total cholesterol, triglyceride, HDL-C, LDL-C), inflammation marker (hs-CRP), and urinalysis. Additionally, vital signs, physical examination, and electrocardiogram will be performed to ensure participant safety.

#### 2.4.4 Baseline and covariate assessments

Dietary intake and physical activity levels will be assessed at baseline and 8 weeks to evaluate potential confounding effects on study outcomes. Participants will be instructed to maintain their usual dietary habits throughout the study period. Dietary intake will be assessed using 24-h dietary records (23) completed on 1 weekday prior to baseline and 8-weeks visits. Physical activity will be measured using the Global Physical Activity Questionnaire (GPAQ) (24), with results quantified as Metabolic Equivalent Task (MET) values (25). These measurements will be used as covariates in additional analyses to adjust for potential baseline differences and changes during the intervention period.

### 2.5 Participant timeline

**FIGURE 1:**
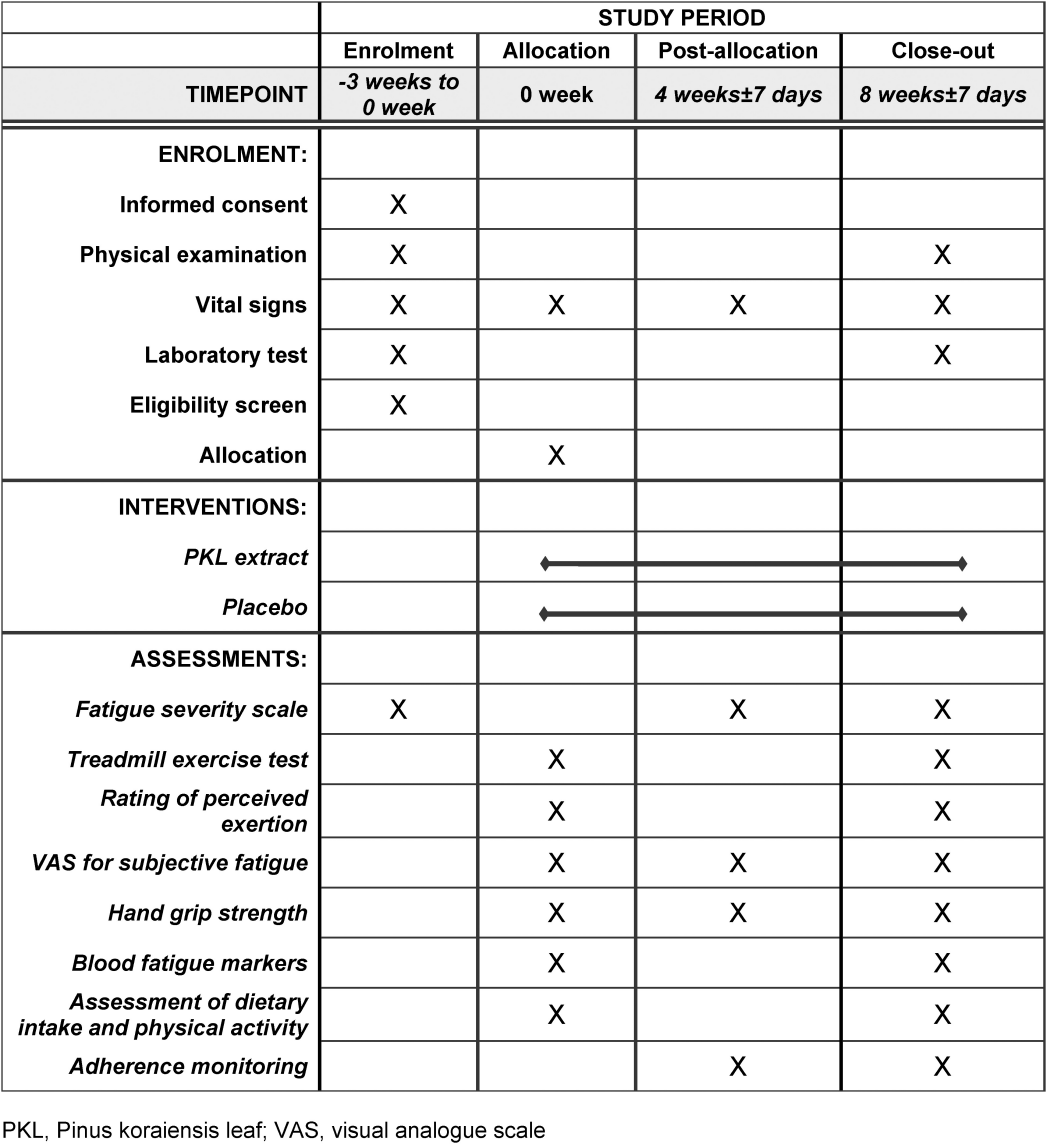
Schedule of enrollment, interventions, and assessments.

The participant timeline is presented in Figure 1.

### 2.6 Sample size

The sample size of this clinical trial is 50 participants per group. Although no preliminary data on PKL extract’s anti-fatigue effects in humans are available, we referenced a study using fermented porcine placental (FPP) extract (26), which has received Korean MFDS approval for fatigue improvement, as PKL extract is pursuing the same regulatory pathway. In that study, the FPP group (*n* = 36) showed a mean change in FSS score of −9.79, while the placebo group (*n* = 31) demonstrated a mean change of −5.20. Based on the reference study data showing a mean difference of 4.59 points in FSS score change between treatment and placebo groups, and assuming a pooled standard deviation of 6.8, we calculated the required sample size. With a significance level of 0.05 and a power of 0.8 for a two-sided test, we estimated that a minimum of 35 participants per group would be required. Considering an anticipated dropout rate of approximately 30% during the clinical trial process, we decided to recruit a total of 100 participants, with 50 participants per group.

### 2.7 Recruitment

Participants will be recruited through IRB-approved recruitment notices posted within and outside the clinical trial institution, website announcements, and newspaper advertisements.

### 2.8 Random allocation and blinding

An independent statistician generated a randomization list using block randomization methods by SAS for Windows, with the block size remaining undisclosed. Access to the randomization list will be strictly limited. PKL extract and placebo are pre-packaged according to the randomization list, and allocation concealment will be maintained by sequentially assigning enrolled participants to randomization numbers. The PKL extract and placebo capsules have identical appearances, ensuring double blinding throughout the study. Blinding will not be broken until all participants complete the trial and the data is locked. Unblinding will only be permitted in emergency situations where knowledge of the intervention is necessary for participant safety, following established procedures. In such cases, the principal investigator must contact the sponsor as promptly as possible and document the date, time, and reason for unblinding.

### 2.9 Data collection methods

The FSS will be administered to assess the level of fatigue experienced by participants during the preceding week. Investigators will ensure that participants fully understand and can properly respond to the questionnaire. The standardized Korean version of the FSS will be used for this study (18). FSS obtained during the screening phase will be used as baseline values.

A treadmill exercise test will be conducted to measure primary outcomes including time and distance to exhaustion, along with secondary outcomes comprising AT, VO2max, RQmax, and HRmax. Additionally, RPE will be assessed at the conclusion of each stage of the treadmill exercise test, immediately before progressing to the next stage.

### 2.10 Data management

Research data will be managed through an electronic case report form (eCRF). In the development of the eCRF, range checks for data values will be implemented for each parameter to minimize the possibility of errors. Investigators will enter study data into the eCRF, and Clinical Research Associates (CRAs) will perform full Source Data Verification (SDV) through regular monitoring of all entered data. Data lock will be executed once verification of all data is completed and preparations for statistical analysis are finalized, including the completion of blind data review meetings for analysis group determination.

### 2.11 Statistical methods

#### 2.11.1 Analysis set

Efficacy data will be primarily analyzed using the Per-Protocol (PP) set, with additional analyses conducted on the Intent-to-Treat (ITT) and Full Analysis (FA) sets. Safety data will be analyzed using the Safety Analysis (SA) set.

The ITT set will include all participants enrolled in this clinical trial. The SA set will comprise subjects who consumed the investigational product at least once after enrollment. The FA set will include all randomized participants with at least one efficacy evaluation after consuming the investigational product. Participants who violated inclusion/exclusion criteria or never consumed the investigational product will be excluded from the FA set. The PP set will consist of participants from the FA set who completed the trial according to the protocol without major violations. Participants who used prohibited concomitant medications or committed other serious protocol violations will be excluded from the PP set. Other serious protocol violations will be determined through a blind data review meeting.

Statistical analyses will be performed using SAS for Windows, with statistical significance set at *p* < 0.05. Missing values will be replaced using the Last Observation Carried Forward (LOCF) method. Baseline characteristics between groups will be compared using Chi-Square test or Fisher’s exact test for categorical variables and Independent *t*-test for continuous variables.

#### 2.11.2 Statistical methods for analyzing efficacy outcomes

For primary and secondary efficacy endpoints, descriptive statistics will be presented by treatment group, and comparisons will be made both between groups.

For between-group comparisons, changes from baseline (post-8-weeks consumption minus pre-consumption) will be analyzed using independent *t*-test if normality assumptions are met, or Mann-Whitney U test if not. The proportion of participants showing improvement in efficacy variables at 8 weeks compared to baseline may be analyzed using Chi-Square test or Fisher’s exact test.

For repeated measurements of FSS (excluding the primary efficacy endpoint analysis), VAS for subjective fatigue, and grip strength (measured three times), RM-ANOVA or Linear Mixed Model will be used for between-group comparisons if normality assumptions are met or appropriate non-parametric methods if not. In cases of severe violation of normality assumptions, variable transformation methods such as logarithm transformation may be applied before analysis.

Additionally, ANCOVA or ranked ANCOVA may be performed with covariates including lifestyle factors (dietary intake, physical activity, alcohol consumption, smoking, occupation, etc.), baseline values and changes in lifestyle factors, baseline values of efficacy variables that are not homogeneous between groups, and demographic information.

#### 2.11.3 Statistical methods for analyzing safety outcomes

All adverse events occurring during the clinical trial period will be summarized as frequencies and percentages by groups. The pattern of adverse events between groups will be analyzed using chi-square test or Fisher’s exact test.

For laboratory test results and vital signs, descriptive statistics will be presented by groups, and between-group comparisons will be conducted. For between-group comparisons, changes from baseline (post-8-weeks consumption minus pre-consumption) will be analyzed using independent *t*-test.

### 2.12 Data monitoring and auditing

Monitoring will be conducted to ensure the protection of participants’ rights and welfare, to verify that reported trial-related data are accurate, complete, and verifiable when compared with source documents, and to confirm that the trial is being conducted in compliance with the approved protocol, ICH and GCP/KGCP guidelines, case report forms, source documents, and applicable regulations.

The monitor will conduct regular site visits and maintain telephone contact with the site. During visits, the monitor will primarily review original participant records, investigational product accountability logs, and research files. Additionally, the monitor will carefully observe the progress of the trial and discuss any issues with the investigator if problems arise.

## 3 Discussion

This clinical trial was designed to evaluate whether the anti-fatigue effects of PKL extract observed in previous animal experiments also occur in humans. The trial was planned in accordance with the Korean MFDS’s functional evaluation guidelines for health functional foods related to fatigue improvement (27), representing a critical step in verifying the potential of PKL extract as a functional food ingredient through human clinical trials.

The anti-fatigue effects of PKL extract are expected to work through several metabolic pathways that will be assessed using consistent biomarkers across both the previous animal experiments and this clinical trial. In mice, PKL extract showed improvements in anaerobic metabolism (reduced blood lactate and LDH activity), protection against muscle damage (reduced CK levels), and enhanced exercise endurance (increased time to exhaustion in swimming and rotarod tests) (9). This clinical trial will measure these identical objective biomarkers in humans to verify whether similar physiological responses occur, while additionally exploring subjective fatigue assessments.

If PKL extract shows significant improvements in both subjective fatigue measures and objective exercise performance parameters consistent with the animal model findings, it will provide evidence for its development as a functional health food ingredient for fatigue improvement. Furthermore, this study aims to generate the necessary data to support the regulatory approval process for PKL extract as a health functional food, bringing this natural product one step closer to benefiting public health.

There are several limitations in this protocol. First, subjective assessment tools such as the Fatigue Severity Scale and Visual Analogue Scale have inherent limitations, though this is mitigated by objective exercise performance measurements and comprehensive fatigue-related biomarkers. Second, the 8-weeks intervention period may be relatively short considering the chronic nature of fatigue (28), and the absence of follow-up limits assessment of sustained effects. Third, our strict inclusion and exclusion criteria, while creating a homogeneous study population, may limit generalizability to broader populations with fatigue, including those with different BMI ranges or chronic conditions. Fourth, as a single-center study, there may be limitations in terms of selection bias and generalizability compared to multi-center trials.

## 4 Ethics and dissemination

### 4.1 Research ethics approval

The protocol of this clinical trial was approved by the Institutional Review Board of Wonkwang University Oriental Medical Hospital, Jeonju (approval number: WUJKMH-IRB-2024-0009).

### 4.2 Protocol amendments

The current version of the protocol is V1.1. If changes to the protocol become necessary, modifications will be made after discussion between the investigators and the sponsor, and will be implemented after obtaining IRB approval.

### 4.3 Consent

Participant consent will be obtained before enrollment in the study. The principal investigator or delegated investigators will explain the content of the clinical trial, expected effects, and safety profile of the investigational product to potential participants according to the attached explanatory document and consent form. Written informed consent will be obtained from those who voluntarily agree to participate in this research. Participants will be given sufficient time to make their decision after receiving the explanation.

### 4.4 Confidentiality

The records identifying the participants will be kept confidential, and the participants’ identities will remain confidential even when the results of the trial are published. All trial-related documents will record and distinguish participants by their identification codes rather than by their names.

### 4.5 Dissemination policy

This clinical trial aims to develop PKL extract as a functional health food ingredient for fatigue improvement. The results of this clinical trial will be submitted to the Ministry of Food and Drug Safety (MFDS) in Korea and published in a peer-reviewed journal.
